# Root triterpenoid metabolites drive the assembly and feedback regulation of the rhizosphere microbiome during flowering to senescence in *Rhododendron hybridum* ‘Yangmeihong’

**DOI:** 10.3389/fmicb.2026.1753104

**Published:** 2026-02-10

**Authors:** Fei Shan, Chenxi Leng, Yufeng Xiao, Ximin Zhang, Ming Tang, Yin Yi, Jing Tang

**Affiliations:** 1 Key Laboratory of State Forestry Administration on Biodiversity Conservation in Karst Area of Southwest, Guizhou Normal University, Guiyang, China; 2 Key Laboratory of Environment Friendly Management on Alpine Rhododendron Diseases and Pests of Institutions of Higher Learning in Guizhou Province, Guizhou Normal University, Guiyang, China

**Keywords:** flowering and senescence, microbial community assembly, rhizosphere microbiome, rhododendron, triterpenoids

## Abstract

The flowering-to-senescence transition is a critical developmental period in ornamental plants, yet the interplay between root metabolites and the rhizosphere microbiome during this process remains poorly characterized. Integrating metabolomic and microbiomic analyses of *Rhododendron hybridum*, we investigated their dynamic interactions. Our analyses revealed both the root metabolome and rhizosphere microbiome exhibited significant temporal dynamics. Differential metabolites significantly enriched in sesquiterpenoid and triterpenoid biosynthesis, while microbial α-diversity peaked at full bloom before declining. The rhizosphere microbial network complexity decreased from flowering to senescence, accompanied by a shift in community assembly from stochastic to more deterministic processes. Furthermore, root metabolites mediated stage-specific assembly of the rhizosphere microbiome. Specifically, seven volatile terpenoids, upregulated during senescence, were significantly correlated with 77 microbial taxa, including putative plant growth-promoting bacteria. Functional prediction suggested that these interacting microbial taxa are potentially involved in sulfur cycling, methionine biosynthesis, and nucleic acid metabolism, indicating a potential role in feedback regulation during senescence. Our findings demonstrate that root triterpenoid metabolites are pivotal in driving rhizosphere microbiome assembly and may receive functional feedback, providing novel insights into microbiome-mediated regulation of floral development and senescence.

## 1 Introduction

Flower opening and senescence are critical developmental stages in the plant life cycle (Rieu et al., 2023). *Rhododendron hybridum* (*R*. *hybridum*), valued for its diverse floral color phenotypes and ornamental traits, is an important horticultural cultivar with significant economic value (Fang et al., 2017). Belonging to the genus *Rhododendron* within the Ericaceae family, it is one of the core species in the global potted flower industry. Its landscape applications span urban greening, garden landscaping, and ecological restoration projects across multiple climatic zones worldwide (De Keyser et al., 2013; Fang et al., 2023; Shrestha et al., 2018). Flowering time is a vital agronomic trait in ornamental plants (Wu T. et al., 2024) whose periodicity and temporal characteristics are positively correlated with commercial value (Sun et al., 2021),. Therefore, modulating the flowering time plays a key role in enhancing the economic value of ornamental plants.

The timing of plant flowering is coordinately regulated by endogenous hormones and environmental signals. Unraveling the molecular mechanisms underlying this process provides a theoretical foundation for the genetic improvement of floral traits (He et al., 2020). As the terminal stage of floral organ development, petal senescence is governed by complex mechanisms, including epigenetic regulation, hormone signal transduction, and transcription factor cascades (Ji et al., 2023; Shrestha et al., 2018; Zhang et al., 2022). Current research on flowering regulation in Rhododendron primarily focuses on phenological observation, functional characterization of key genes, and the interaction between internal and external signaling pathways (Cheon et al., 2011; Wang et al., 2018; Zhang et al., 2022). In contrast, the regulatory mechanisms underlying flower senescence in this genus remain less explored, with existing achievements largely centered on the identification and study of key senescence-associated genes (Fang et al., 2023; Xu et al., 2022). Notably, recent evidence suggests that the rhizosphere microbiome can influence flowering time by modulating the balance of endogenous plant hormones (Lu et al., 2018); however, its regulatory role in the flower senescence process awaits further elucidation.

Plants within the genus *Rhododendron* have evolved distinct secondary metabolic profiles as part of their chemical defense and ecological adaptation strategies. This genus is characterized by its richness in grayanotoxins, signature flavonoids, and triterpenoids (Łyko et al., 2024; Peng et al., 2020; Wen et al., 2025; Yang et al., 2024). These metabolites not only mediate plant defense against herbivorous insects and pathogenic microorganisms but, more crucially, can be secreted into the rhizosphere via root exudates. There, they exert strong selective pressures, actively shaping the structure of the rhizosphere microbiome (Bais et al., 2006). Therefore, integrating the inherent root chemical background of Rhododendron into the research framework is a key prerequisite for elucidating the mechanisms underlying its rhizosphere microecological interactions. Based on this chemical rationale, this study focuses on the dynamics of terpenoids and other metabolites during floral transition. It aims to decipher how the plant developmental program drives adaptive reorganization of the microbial community through specific metabolites, which represents a frontier approach in the field.

Within this chemical framework, root-secreted terpenoids, particularly triterpenoids, play a pivotal role. Studies have shown that triterpenoids in the rhizosphere can function simultaneously as antimicrobial agents suppressing pathogens, as signaling molecules mediating symbiotic dialogue, and as specific carbon sources selectively enriching microbial taxa capable of metabolizing them (Huang et al., 2019; Nakamura and Sugiyama, 2025). Notably, the functions of the broader terpenoid metabolic network extend far beyond these roles. For instance, strigolactones—derived from carotenoids (a type of tetraterpenoid)—act as crucial root-derived signaling molecules and plant hormones. They play a central role in regulating plant development, symbiosis, and senescence processes, with their mutants often exhibiting a delayed senescence phenotype (Clark and Bennett, 2024; Ur Rehman et al., 2021; Wang X. et al., 2024). This important insight reveals that the functional repertoire of terpenoid metabolic pathway products, including those from its upstream triterpene synthesis branches, has profoundly expanded beyond traditional direct defense. These compounds are now understood to be integral to modulating plant developmental timing and co-evolving with the rhizosphere microbiome. This provides a coherent and compelling theoretical foundation for our study’s focus on the dynamics of terpenoids—specifically triterpenoids—during floral transition, positioning them as potential core drivers of rhizosphere microbiome restructuring.

Dynamic plant-rhizosphere microbiome interactions play crucial roles in regulating plant growth, development, and stress resistance (Geller and Levy, 2023). In *Brassica napus*, key developmental stages such as bolting and silique development significantly alter the alpha diversity of the rhizosphere microbiome (Wang M. et al., 2024; Yadav et al., 2023). Chaparro et al. (2014) demonstrated that the rhizosphere microbiome of the model plant *Arabidopsis thaliana* (*A*. *thaliana*) exhibits clear temporal dynamics during development, with distinct microbial community compositions between the seedling stage and other developmental phases (Haichar et al., 2016; Mahmud et al., 2020). Plants recruit stage-specific microbes through temporal changes in root metabolites, and these selectively recruited microbes subsequently influence plant growth and development. For example, plant growth-promoting rhizobacteria can modulate root architecture remodeling through relevant signaling pathways (Li et al., 2024; Lyu et al., 2021). Microbial transplantation experiments in *A*. *thaliana* have shown that inoculation with rhizosphere microbiota from different Flowering Stage (FS) sources during the FS of *A*. *thaliana* can lead to either accelerated or delayed flowering time (Panke-Buisse et al., 2015). These findings collectively highlight an inseparable relationship between the rhizosphere microbiome and plant flowering. However, research on the reciprocal interactions with the flowering process remains relatively limited, which significantly constrains the development of technical frameworks for manipulating flowering process via the rhizosphere microbiome.

The transition between plant developmental stages is accompanied by alterations in metabolic cycles, such as carbohydrate and energy metabolism, which consequently influence the composition of root metabolites. Thus, root metabolites vary across different developmental stages of the plant (Lu et al., 2021; Yue et al., 2023). For instance, studies in *A*. *thaliana* have demonstrated that the chemical profiles of root exudates are precisely regulated by the developmental program. Furthermore, these dynamic metabolite secretion patterns directly determine which microbial taxa can colonize and proliferate in the rhizosphere, thereby driving the stage-specific assembly of the microbial community (Badri et al., 2010; Zhalnina et al., 2018). As chemical hubs in the regulation of plant development, root metabolites comprise diverse chemical classes, including glycosides, phenylpropanoids, terpenoids, flavonoids, and nitrogen-containing secondary metabolites, which collectively participate in modulating plant growth and development (Afridi et al., 2024; Wang et al., 2023). During the flowering process, root secretions of defense-related proteins such as chitinases are significantly enhanced. The coordinated transport of these root-derived defense proteins and nutrients provides dual support for floral organ development (De-la-Peña et al., 2010).

Root metabolites mediate plant-rhizosphere microbiome interactions: plants shape microbial communities through secretion, while microbes in turn regulate metabolite composition via root-associated signaling (Korenblum et al., 2020; Sasse et al., 2018; Spaepen et al., 2007). In Arabidopsis, certain microbes convert root-derived tryptophan to indole-3-acetic acid (IAA), which delays flowering by downregulating flowering-related genes (Lu et al., 2018). Despite advances in understanding plant development, root metabolites, and the rhizosphere microbiome, key questions in Rhododendron remain unresolved: (i) dynamic patterns of root metabolites during flowering, (ii) succession of the rhizosphere microbial community, and (iii) interactions between root metabolites and the microbiome. This gap limits the design of flowering-manipulation strategies in ornamental rhododendrons.

Based on this integrated framework, we propose a mechanism-focused hypothesis: during the process from flowering to senescence in *R. hybridum*, the biosynthesis profile of root-derived triterpenoids undergoes an orderly reprogramming. Specifically, certain triterpenoid metabolites that accumulate preferentially during the senescing stage. act as key drivers actively shaping the rhizosphere microbial community. Through antimicrobial, signaling, and/or nutritive functions, these compounds selectively inhibit certain microbial taxa while enriching functional groups that may assist in nutrient recycling or alleviate senescence-associated stress, thereby “assembling” a specific rhizosphere microenvironment that serves the senescence process. In turn, this remodeled microbial community may further influence the host’s senescence process via feedback regulation, for instance, by modulating phytohormone dynamics or stress responses.

This study utilized 3-year-old potted *R. hybridum* as experimental material. We collected root tissue and rhizosphere soil samples from five critical stages spanning from flowering to senescence (bud stage (TB), initial blooming stage (TIB), full bloom stage (TFB), senescence stage (TS), and fading stage (TF)). The microbial community structure was analyzed via 16S rRNA high-throughput sequencing. Dynamic changes in root metabolites were captured using ultra-high-performance liquid chromatography tandem mass spectrometry (UPLC-MS/MS) and gas chromatography-mass spectrometry (GC-MS). This study aims to systematically elucidate: (i) whether the dynamic changes in the root metabolome—particularly in triterpenoid compounds—are coupled with the assembly and succession of the rhizosphere microbiome across the temporal progression from flowering to senescence; (ii) to reveal the interactions between root-derived triterpenoid metabolites and the rhizosphere microbial community during the flowering-to-senescence transition; and (iii) to identify the core functional microbial taxa that may play key roles during floral senescence and to preliminarily investigate their potential functional mechanisms, with a specific focus on their associations with triterpenoid metabolites.

## 2 Materials and methods

### 2.1 Plant sample collection

The plant materials, *R. hybridum* ‘Yangmeihong,’ were obtained from a commercial nursery in Huishui County, Guizhou Province. We selected uniform, healthy, 3-year-old potted plants that were free from pests and diseases. At the time of selection, all plants were at the pre-bud stage (petals completely enclosed by sepals). Plants were uniformly cultivated in a greenhouse under the following conditions: a 16-h/8-h light/dark cycle, temperature of 22°C, light intensity of 400 μmol⋅m^–2^⋅s^–1^, and relative humidity of 60–70%. Based on the floral developmental status of potted *R. hybridum* plants, the process from flowering to senescence was divided into five consecutive phenological stages for sampling: (i) TB (Bud stage): Over 80% of the flowers were unopened red buds. (ii) TIB (Initial Bloom stage): Over 80% of the petals began to unfurl but remained semi-closed. (iii) TFB (Full Bloom stage): Over 80% of the petals were fully open. (iv) TS (Senescence Stage): Over 80% of the petals began to wilt and turned dark red. (v) TF (Fading Stage): Over 80% of the petals were completely wilted, appearing dark purple, with the perianth separating from the pedicel (Supplementary Figure S1). Sampling was conducted immediately once the plants in each stage met the above morphological criteria. The period from the current stage to the next stage was defined as the time interval required to reach the respective criteria (i.e., the duration of that stage). Specifically, these intervals were approximately: TB to TIB: 17 days; TIB to TFB: 9 days; TFB to TS: 25 days; TS to TF: 9 days.

### 2.2 Rhizosphere soil collection

The entire plant was carefully removed from the pot. Loosely attached soil, defined as bulk soil, was removed by gently shaking the root system. The soil tightly adhering to the root surface was retained and defined as rhizosphere soil. The root system with adhering soil was then placed into a sterile plastic bag and vigorously shaken to collect the rhizosphere soil. All collected samples were immediately flash-frozen in liquid nitrogen and stored at −80°C for subsequent analysis. The experiment encompassed all five developmental stages, with three biological replicates per stage.

### 2.3 Root tissue collection

Following rhizosphere soil collection, the root systems were excised from the plants. Residual soil was removed by thoroughly rinsing the roots with sterile ultrapure water. The cleaned roots were then sealed in sterile plastic bags, flash-frozen in liquid nitrogen, and stored at −80°C for subsequent analysis. Three biological replicates were also prepared for each stage.

### 2.4 UPLC-MS/MS-based analysis of secondary root metabolites

Freeze-dried samples were ground for 1.5 min at 30 Hz using a mixer mill (Retsch MM 400). Precisely 50 mg of the resulting powder was weighed, and 1,200 μL of a pre-cooled (4°C) 70% aqueous methanol solution containing internal standards was added. The samples were vortexed for 30 s, with this process repeated every 30 min for a total of six cycles, followed by centrifugation at 12,000 rpm for 3 min at 4°C. The supernatant was collected, filtered through a 0.22 μm microporous membrane, and transferred into vials for analysis. Metabolites analysis was performed using an ultra-performance liquid chromatography-triple quadrupole tandem mass spectrometry (UPLC-QQQ-MS/MS) system. Chromatographic separation was achieved using an Agilent SB-C18 column (1.8 μm, 2.1 × 100 mm) maintained at 40°C, with a flow rate of 0.35 mL/min. Mobile phase A consisted of water with 0.1% formic acid, and mobile phase B was acetonitrile with 0.1% formic acid. The elution gradient was programmed as follows: 0 min (5% B) → 9.0 min (95% B) → 10.0 min (95% B) → 11.1 min (5% B) → 14.0 min (5% B). Mass spectrometric detection was conducted using an electrospray ionization source, with data acquired in both positive and negative ion modes. The ion source temperature was set at 500°C, with spray voltages of +5,500 V and −4500 V for positive and negative modes, respectively. Curtain gas, nebulizer gas, and auxiliary gas pressures were set at 25, 50, and 60 psi, respectively, with nitrogen as the collision gas. Metabolite identification was based on multiple reaction monitoring, screening for precursor ions and characteristic fragment ions to exclude matrix interference. Metabolites were annotated by matching secondary mass spectra against a self-built metabolite database (MWDB), excluding signals from isotopes, adduct ions (e.g., K^+^, Na^+^, NH_4_^+^), and duplicate fragments (Fraga et al., 2010). Quantitative analysis utilized MRM mode to monitor characteristic ion pairs, with declustering potential and collision energy optimized for enhanced detection specificity. Chromatographic peak identification and integration were performed using Analyst 1.6.3 and MultiQuant 3.0.3 software (Chen et al., 2013). Peak alignment across samples was conducted based on retention time and peak shape, with metabolite relative levels expressed as peak areas.

### 2.5 GC-MS-based analysis of volatile root metabolites

Frozen samples were ground in liquid nitrogen, and 500 mg of the resulting powder was precisely weighed into a 20 mL headspace vial. A saturated sodium chloride solution and 10 μL of internal standard solution (50 μg/mL) were added. Volatile compounds were enriched using fully automated headspace solid-phase microextraction with a 120 μm DVB/CWR/PDMS SPME Arrow fiber, which provides approximately 10-fold higher sensitivity than conventional fibers. The samples were equilibrated at 60°C for 5 min with continuous agitation, followed by a 15 min headspace extraction, and finally thermal desorption in the injection port at 250°C for 5 min. New SPME Arrow fibers were conditioned at 250°C for 2 h prior to initial use, and for 5 min at the same temperature before each subsequent analysis. GC-MS analysis was performed under the following conditions: chromatographic separation used a DB-5MS capillary column (30 m × 0.25 mm × 0.25 μm) with high-purity helium (purity ≥ 99.999%) as the carrier gas at a constant flow rate of 1.2 mL/min. The injector temperature was set at 250°C in splitless mode with a 3.5 min solvent delay. The temperature program was: initial temperature 40°C held for 3.5 min, ramped to 100°C at 10°C/min, then to 180°C at 7°C/min, and finally to 280°C at 25°C/min with a 5 min hold. Mass spectrometric detection employed an electron ionization source at 70 eV, with ion source temperature 230°C, quadrupole temperature 150°C, and transfer line temperature 280°C. Data acquisition used selected ion monitoring mode according to Chinese National Standard GB 23200.8-2016. Metabolite identification was performed by matching mass spectral characteristic ions and retention times against a self-built database (Yuan et al., 2022). Raw data were processed using MassHunter software for peak integration and calibration of target compounds. Quantitative analysis was based on the internal standard method, calculating relative metabolite levels according to characteristic ion peak areas. To ensure data quality, multiple measures were implemented: internal standards were included in each batch to monitor extraction efficiency; blank samples were analyzed regularly to exclude background interference; SIM mode was used to improve signal-to-noise ratio; and retention time shift correction was applied to ensure cross-sample comparability.

### 2.6 Microbial genomic DNA extraction and full-length 16S rRNA gene sequencing

Total microbial genomic DNA was extracted from frozen rhizosphere soil samples using the PowerSoil DNA Isolation Kit. The full-length region of the 16S rRNA gene was amplified using universal primers 27F (5′-AGRGTTTGATYNTGGCTCAG-3′) and 1492R (5′-TASGGHTACCTTGTTASGACTT-3′), which were supplemented with sample-specific barcodes. The PCR products were purified, and their concentration and purity were assessed using a NanoDrop spectrophotometer (Thermo Scientific, United States). Purified amplicons were normalized to an equal concentration for the construction of SMRTbell libraries. After quality control, single-molecule real-time (SMRT) sequencing was performed on the PacBio Sequel II system (Pacific Biosciences, United States) using the circular consensus sequencing mode to obtain high-quality reads. Raw circular consensus sequencing sequences were generated using the PacBio SMRT Link toolkit. Subsequently, sample barcodes were identified using lima v1.7.0, and primer sequences were trimmed, with length filtering applied, using cutadapt v1.9.1. Chimeric sequences were removed using UCHIME v4.2, resulting in high-quality effective circular consensus sequencing reads. These sequences were clustered into operational taxonomic units (OTUs) at 99.9% similarity using Usearch (Zhou et al., 2024). Representative sequences from each OTU were taxonomically classified against the Silva database using a naïve Bayesian classifier, and the resulting taxonomic assignments were further processed and analyzed with QIIME software.

### 2.7 Microbial co-occurrence network construction and topological feature analysis

To reduce sequencing noise and exclude the interference of low-abundance microbes in network construction, the raw microbial abundance table underwent a two-step filtering process. First, microbial abundances across all samples were converted to relative abundances via total-sum scaling. The average relative abundance of each microbe across all samples was then calculated, and those with an average relative abundance below 0.01% were removed. Subsequently, microbes present in fewer than 1/6 of the samples were further filtered out (Weiss et al., 2017), resulting in 1,640 microbes retained for subsequent analysis. Based on the filtered relative abundance table, a microbial co-occurrence network was constructed using Spearman’s correlation, with thresholds set at |*r*| > 0.7 and a false discovery rate corrected *p* < 0.05 (Jiao et al., 2022). Network visualization was performed using Gephi 0.10.1 (Pereira et al., 2021). Topological properties of the network, including the number of nodes, number of links, network diameter, relative modularity (RM), average clustering coefficient, and average degree, were calculated using the igraph package in R (Shen et al., 2023). To further assess the stability of the microbial co-occurrence network, robustness was evaluated from three perspectives: random node removal, targeted attack on keystone nodes, and natural connectivity (Wu L. et al., 2024). All metrics were computed and visualized using the R package ggClusterNet.

### 2.8 Analysis of microbial community assembly processes and the influence of root metabolites

To determine the relative contributions of deterministic and stochastic processes in rhizosphere microbial community assembly, a null model-based analysis incorporating phylogenetic structure was employed. First, microbes were retained if they met either of the following criteria: (1) having a relative abundance ≥ 1 in at least two samples within a group, or (2) being present (abundance ≥ 0) in at least one sample and having an abundance ≥ 1 in another sample. This filtering criterion ensured the inclusion of microbes with significant presence while excluding those with very low or sporadic occurrence. Based on the filtered microbial abundance table and the phylogenetic tree, the β-nearest taxon index (βNTI) and the Raup-Crick index based on Bray-Curtis dissimilarity (RCbray) were calculated between sample pairs. βNTI quantifies the phylogenetic turnover, whereas RCbray reflects community composition differences unrelated to phylogeny. Furthermore, two types of null models were constructed to generate 1,000 random communities: to estimate βNTI, microbial labels were randomly shuffled across the phylogenetic distance matrix; to estimate RCbray, microbes were randomly reassigned while maintaining species occurrence frequency and sample total abundance. Using thresholds of |βNTI| > 2 and |RCbray| > 0.95, the community assembly processes were categorized into five ecological processes: homogeneous selection, heterogeneous selection, dispersal limitation, homogenizing dispersal, and drift (Xiong et al., 2021; Zhou and Ning, 2017). The aforementioned analysis of microbial community assembly processes and the statistical assessment of root metabolite influences were performed in the R environment, primarily utilizing packages such as “picante,” “ape,” “parallel,” “tibble,” “dplyr,” “vegan,” “ggplot2,” and “ggpubr” (Wen et al., 2022).

### 2.9 Statistical analysis

Screening of differentially accumulated metabolites (DAMs) was performed on the MetaboAnalyst 6.0 platform,^1^ using criteria of variable importance in projection > 1 and |log2(Fold Change)| ≥ 1 (Chong and Xia, 2018). Principal component analysis, Venn diagrams, Short Time-Series Expression Miner (STEM) analysis, and KEGG enrichment analysis were conducted using the OmicShare tools platform.^2^ Differences in microbial community structure were assessed via principal coordinates analysis (PCoA) based on weighted UniFrac distance and permutational multivariate analysis of variance (PERMANOVA); differential abundance analysis using edgeR and the generation of species composition stacked bar plots were also completed on the OmicShare platform. Community richness and diversity were characterized by the Chao1 index and Simpson index, respectively. These indices were calculated in the R environment (v4.3.3) using the vegan package (v2.6-8). Differences in alpha diversity indices among groups were statistically compared using one-way analysis of variance followed by Tukey’s HSD *post-hoc* test in SPSS 19.0, with results presented as bar charts displaying significance markers. The association between root metabolites and microbial community structure was tested using the Mantel test (vegan package), based on Bray-Curtis and Euclidean distance matrices with 999 permutations. A metabolite-microbe correlation network was constructed using Spearman’s correlation coefficient (|*r*| > 0.7, *p* < 0.05) and visualized on the OmicStudio cloud platform.^3^

## 3 Results

### 3.1 Generating metabolomic and microbiomic resources for *R. hybridum*

**FIGURE 1:**
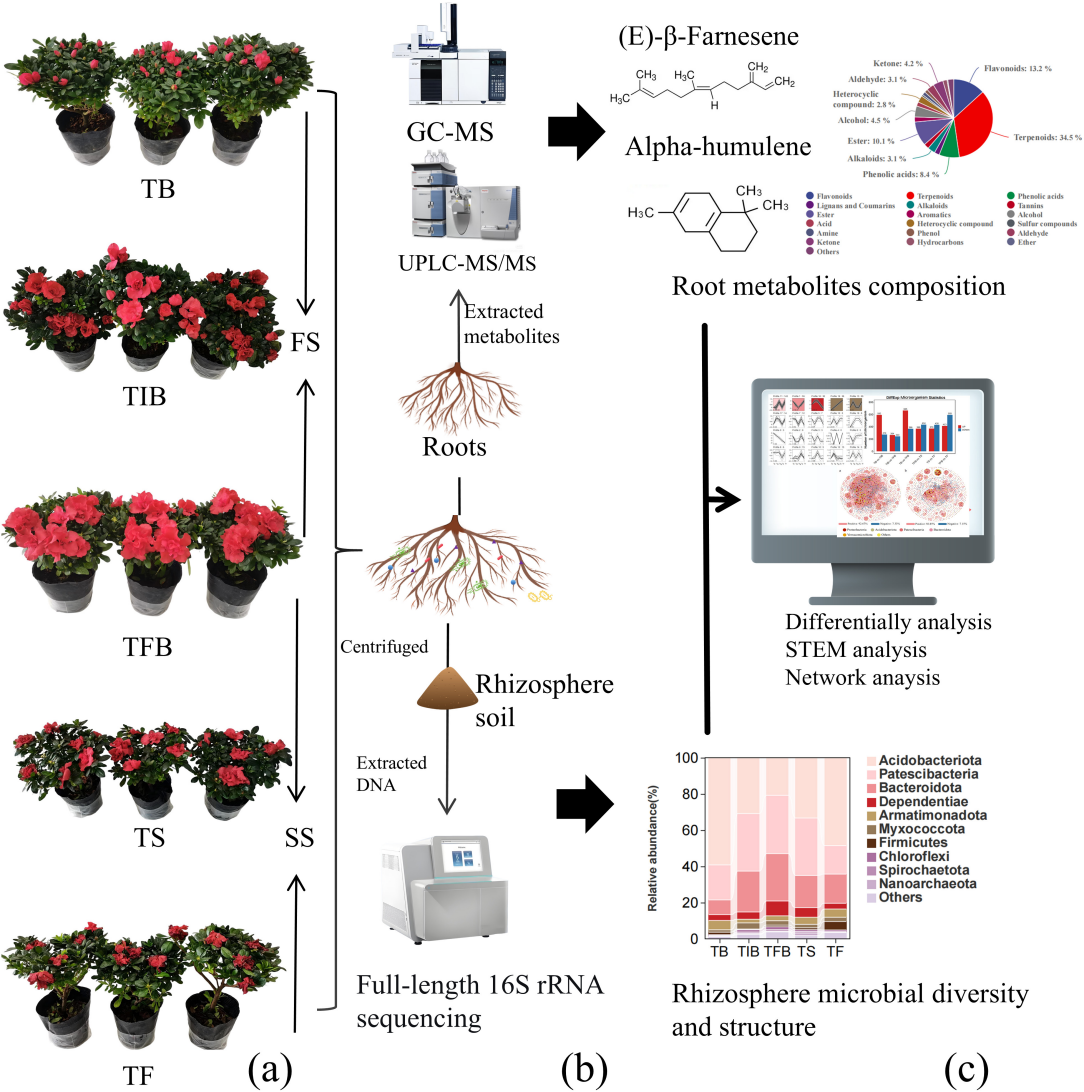
Overview of the experimental design and analysis. **(a)** Sampling stages and grouping. **(b)** Root tissue and rhizosphere soil treatment with metabolomics detection and microbiome sequencing. **(c)** Bioinformatics and statistical analysis pipeline. TB, Bud Stage; TIB, Initial Blooming Stage; TFB, Full Bloom Stage; TS, Senescence Stage; TF, Fading Stage. GC-MS, gas chromatography-mass spectrometry; UPLC-MS/MS, ultra-performance liquid chromatography-tandem mass spectrometry.

To investigate the changes in root metabolites and the rhizosphere microbiome during the flowering process of *R*. *hybridum*, this study used 3-year-old plants and divided the complete process from flowering to senescence into five key stages: TB, TIB, TFB, TS, and TF. Among these, FS encompassed TB, TIB, and TFB, while the SS included TFB, TS, and TF (Figure 1a). Root system samples were collected at each stage and separated into root tissue and rhizosphere soil. For root metabolite analysis, a combination of UPLC-MS/MS and GC-MS techniques was employed for the analysis of metabolites in the root tissue. For rhizosphere microbial community analysis, third-generation sequencing technology was applied to resolve the microbial community structure in the rhizosphere soil (Figure 1b). Subsequent bioinformatics analyses, including differential analysis, STEM analysis, and co-occurrence network analysis, were performed on the generated data to reveal the dynamic interactions between metabolites and the microbial community during the flowering process (Figure 1c). Untargeted metabolomic analysis via GC-MS and UPLC-MS/MS identified a total of 1,429 metabolites in root samples across the five key flowering stages (TB-TF), comprising 271 phenolic acids, 234 flavonoids, 82 alkaloids, among others (Supplementary Table S1). Based on full-length 16S rRNA gene sequencing analysis of rhizosphere microbial DNA across the five stages from flowering to senescence, a total of 5,719 OTUs were obtained, annotated to 28 phyla and 408 species (Supplementary Tables S2, S3). At the phylum level, for example, Proteobacteria, Acidobacteriota, and Patescibacteria were the relatively high-abundance groups. Detailed annotation information at the species level is provided in Supplementary Table S3.

### 3.2 Changes in root metabolites from flowering to senescence in *R. hybridum*

**FIGURE 2:**
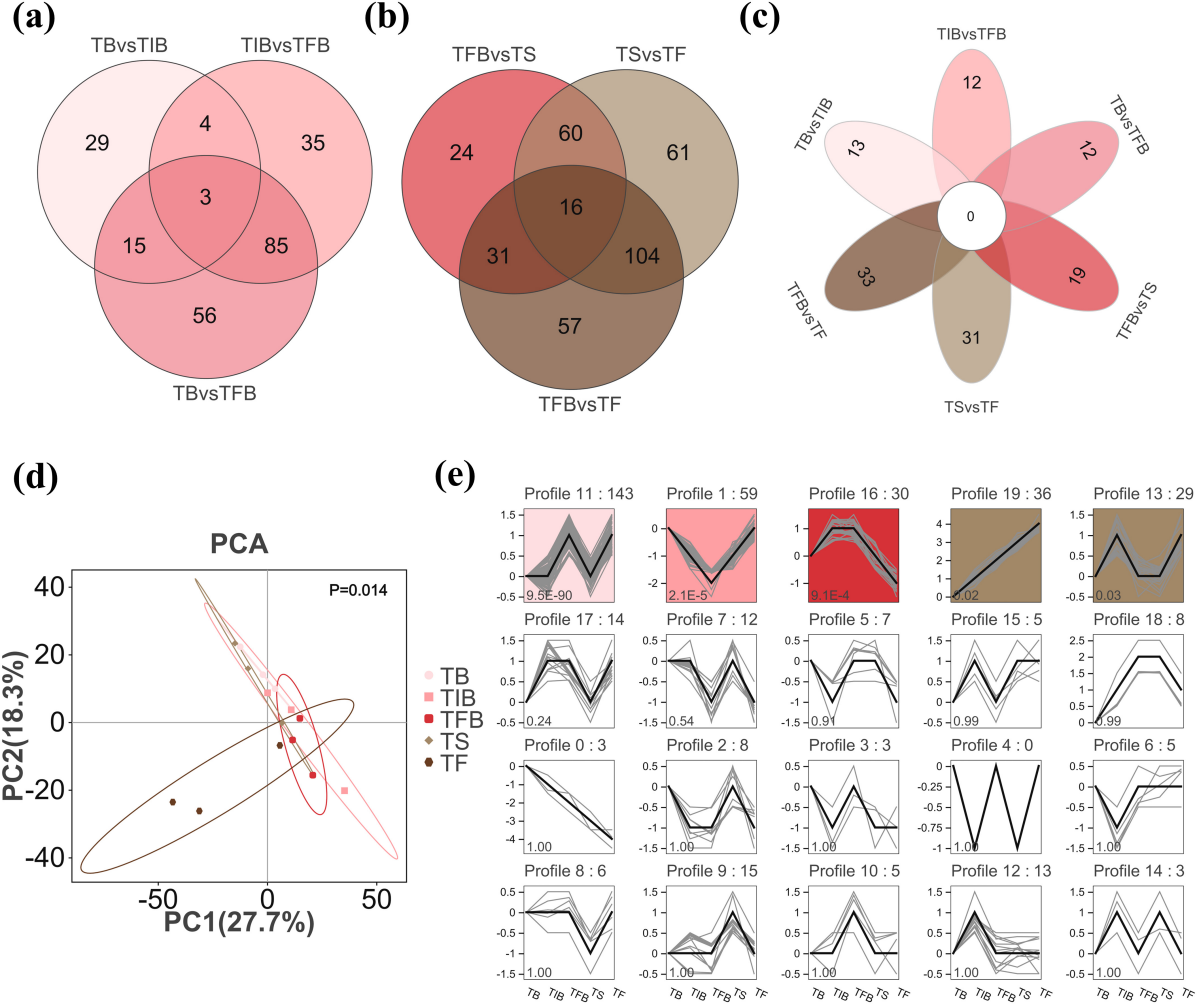
Dynamic analysis of DAMs during the flowering to senescence stages in *R. hybridum*. **(a)** Venn diagram of DAMs in the flowering stage. **(b)** Venn diagram of DAMs in the senescing stage. **(c)** Petal Venn diagram of DAMs from six pairwise comparisons. **(d)** Distribution of root metabolites in the PCA score plot. **(e)** STEM analysis of the 404 DAMs across the flowering to senescence process. TB, Bud Stage; TIB, Initial Blooming Stage; TFB, Full Bloom Stage; TS, Senescence Stage; TF, Fading Stage; STEM, Short Time-Series Expression Miner.

Untargeted metabolomic analysis using GC-MS and UPLC-MS/MS identified a total of 1,429 metabolites in root samples across the five key flowering stages (TB to TF) (Supplementary Figure S2). Orthogonal partial least squares-discriminant analysis revealed 404 DAMs during the progression from flowering to senescence. Among these, 227 DAMs were identified in the FS (Figure 2a), and 353 were found in the SS (Figure 2b). No DAMs persisted consistently throughout the entire process from flowering to senescence (Figure 2c). KEGG pathway enrichment analysis of the 404 DAMs showed significant enrichment solely in the sesquiterpenoid and triterpenoid biosynthesis pathway (*p* = 1.95 × 10^−3^) during the transition from FS to SS. This enriched pathway included seven volatile terpenoids detected by GC-MS: (E)-beta-farnesene, beta-selinene, alpha-humulene, delta-cadinene, alpha-farnesene, (Z)-1-Methyl-4-(6-methylhept-5-en-2-ylidene)cyclohex-1-ene, and (E)-1-Methyl-4-(6-methylhept-5-en-2-ylidene)cyclohex-1-ene (Supplementary Figure S3A; Supplementary Table S4).

The number of stage-specific DAMs was higher in SS than in FS (83 vs. 37). Stage-specific DAMs accounted for 30.8% of the total in FS and 69.2% in SS (Figure 2c). Principal component analysis combined with PERMANOVA confirmed significant differences in the root metabolome across the five flowering stages (*p* = 0.014) (Figure 2d). Therefore, we speculated that root metabolites differ between the flowering and senescing stages, and that flower development and senescence influence root metabolite content. Furthermore, STEM analysis was employed to identify the dynamic patterns of DAMs throughout the flowering-to-senescence process. The results revealed five statistically significant temporal expression patterns (*p* < 0.05) out of 20 total clusters, encompassing 297 DAMs. Specifically, Profile 11 (*n* = 143) metabolites peaked at TFB, decreased to baseline at TS, and re-accumulated at TF. Profile 1 (*n* = 59) metabolites maintained low levels from TB to TFB and returned to their initial levels from TFB to TF. Profile 16 (*n* = 30) showed a significant up-regulation from TB to TIB, stabilized from TIB to TFB, and then gradually decreased until TF. Profile 19 (*n* = 36) displayed a continuous linear increasing trend from TB to TF. Profile 13 (*n* = 29) exhibited a “W”-shaped dynamic: it increased from TB to TIB, then decreased into TFB, remained stable until TS, and increased again from TS to TF (Figure 2e). Among the 297 DAMs showing statistically significant temporal expression patterns from FS to SS, 129 were secondary metabolites, including 38 flavonoids, 28 terpenoids, and 24 phenolic acids, while 168 were volatile metabolites, comprising 71 terpenoids and 29 esters (Supplementary Table S5). Terpenoids were the most abundant class, collectively accounting for 34.5% of the total significant DAMs (Supplementary Figure S3B).

### 3.3 Temporal dynamics of the rhizosphere microbiome in response to the flowering-to-senescence process

**FIGURE 3:**
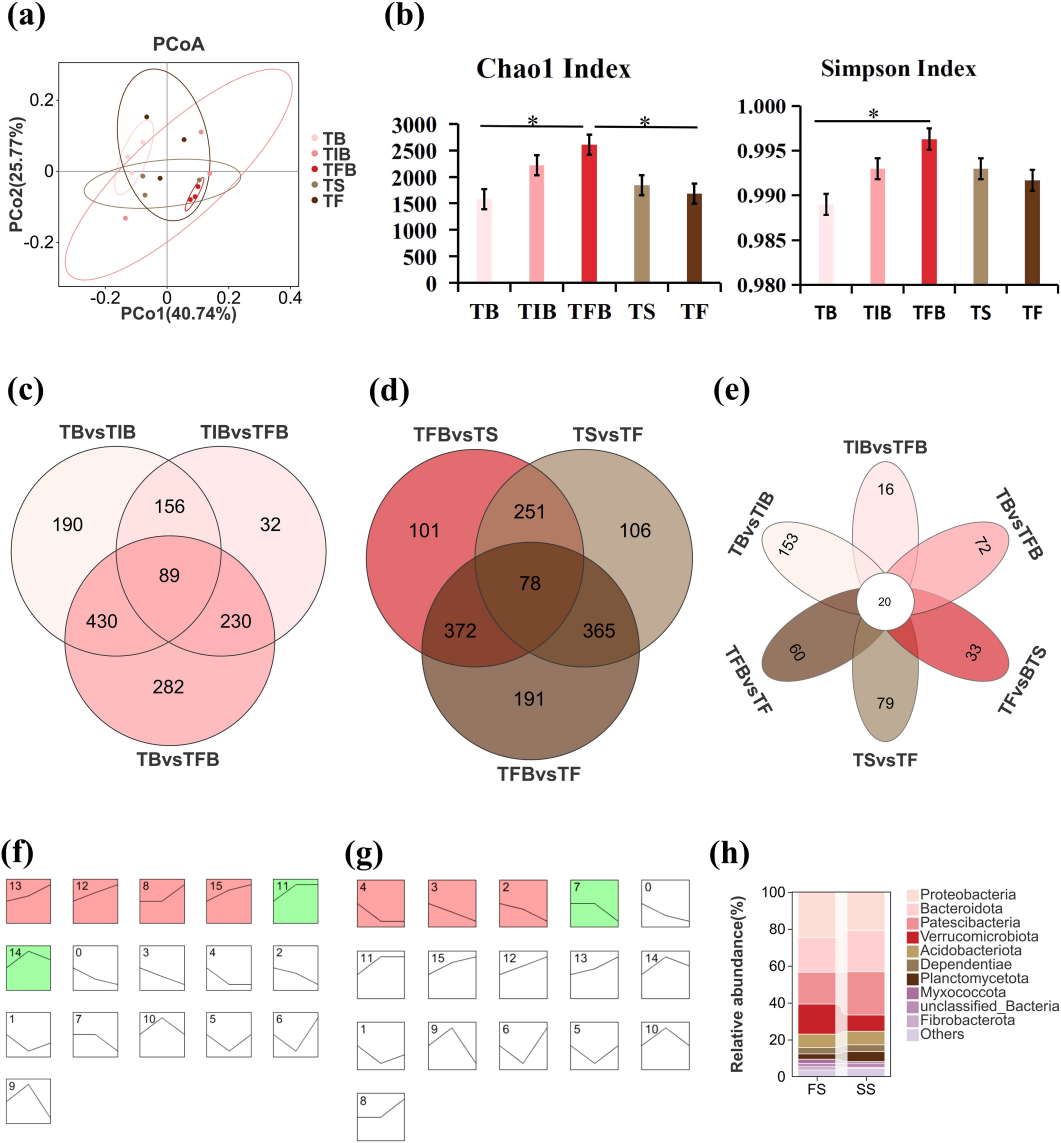
Dynamic changes in the rhizosphere microbial community of *R. hybridum* from flowering to senescence stages. **(a)** PCoA plot based on weighted UniFrac distance, showing the differences in microbial community structure among the five stages. **(b)** Bar charts of the Chao1 index and Simpson index. Error bars represent standard deviation (*n* = 3). Significant differences are indicated by asterisks (*p < 0.05). **(c–e)** Venn diagrams displaying the overlap and uniqueness of differentially abundant OTUs among different comparison groups: **(c)** flowering stage, **(d)** senescing stage, and **(e)** all six pairwise comparisons. **(f,g)** STEM analysis of differential OTUs in the **(f)** flowering stage and **(g)** senescing stage. Colored profiles represent OTUs with statistically significant trends (*p* < 0.05). **(h)** Relative abundance of the top 10 phyla among the significantly differential OTUs. FS, Flowering Stage; SS, Senescing Stage; TB, Bud Stage; TIB, Initial Blooming Stage; TFB, Full Bloom Stage; TS, Senescence Stage; TF, Fading Stage; STEM, Short Time-Series Expression Miner.

Principal coordinates analysis (PCoA) based on weighted UniFrac distance revealed that 66.51% of the variation in the microbial community (PCoA1 + PCoA2) across the flowering to senescence process could be attributed to floral stage succession (Figure 3a). Concurrently, PERMANOVA confirmed that the flowering to senescence process significantly influenced rhizosphere microbiome structure (*p* = 0.021). The richness (Chao1 index) and diversity (Simpson index) of the rhizosphere microbial community exhibited a hump-shaped trend, increasing initially and then decreasing during the progression from flowering to senescence (Figure 3b). Both community richness and diversity at the TB and TF were significantly lower than those at the TFB. The highest microbial richness and diversity throughout the entire flowering process were observed at TFB (Supplementary Table S6). These results demonstrate that the diversity and composition of the Rhododendron rhizosphere microbiome were significantly altered across the five floral stages, with community richness and evenness increasing during the FS and subsequently decreasing during the SS, peaking at the TFB.

Full-length 16S rRNA gene sequencing of rhizosphere microbial DNA across the five floral stages yielded 5,719 OTUs, which were taxonomically assigned to 28 phyla and 408 species. A total of 1,409 differential OTUs were identified in the FS (Figure 3c), and 1,464 differential OTUs were found in the SS (Figure 3d). Across the entire flowering-to-senescence process, 2,010 differential OTUs were identified (Figure 3e). Furthermore, STEM analysis was applied to cluster these differential OTUs based on their temporal patterns. The analysis identified 589 and 374 OTUs with statistically significant temporal changes in the FS and SS, respectively, which were grouped into 6 and 4 distinct expression profiles. The significant OTUs in the FS generally exhibited an increasing trend (Figure 3f), while those in the SS were predominantly characterized by a decreasing trend (Figure 3g). At the phylum level, these significant differential microbes from both FS and SS were primarily enriched in five major phyla: Proteobacteria, Patescibacteria, Bacteroidota, Verrucomicrobiota, and Acidobacteriota (Figure 3h).

### 3.4 Influence of flowering and senescing stages on rhizosphere microbial co-occurrence network complexity and keystone microbes

**FIGURE 4:**
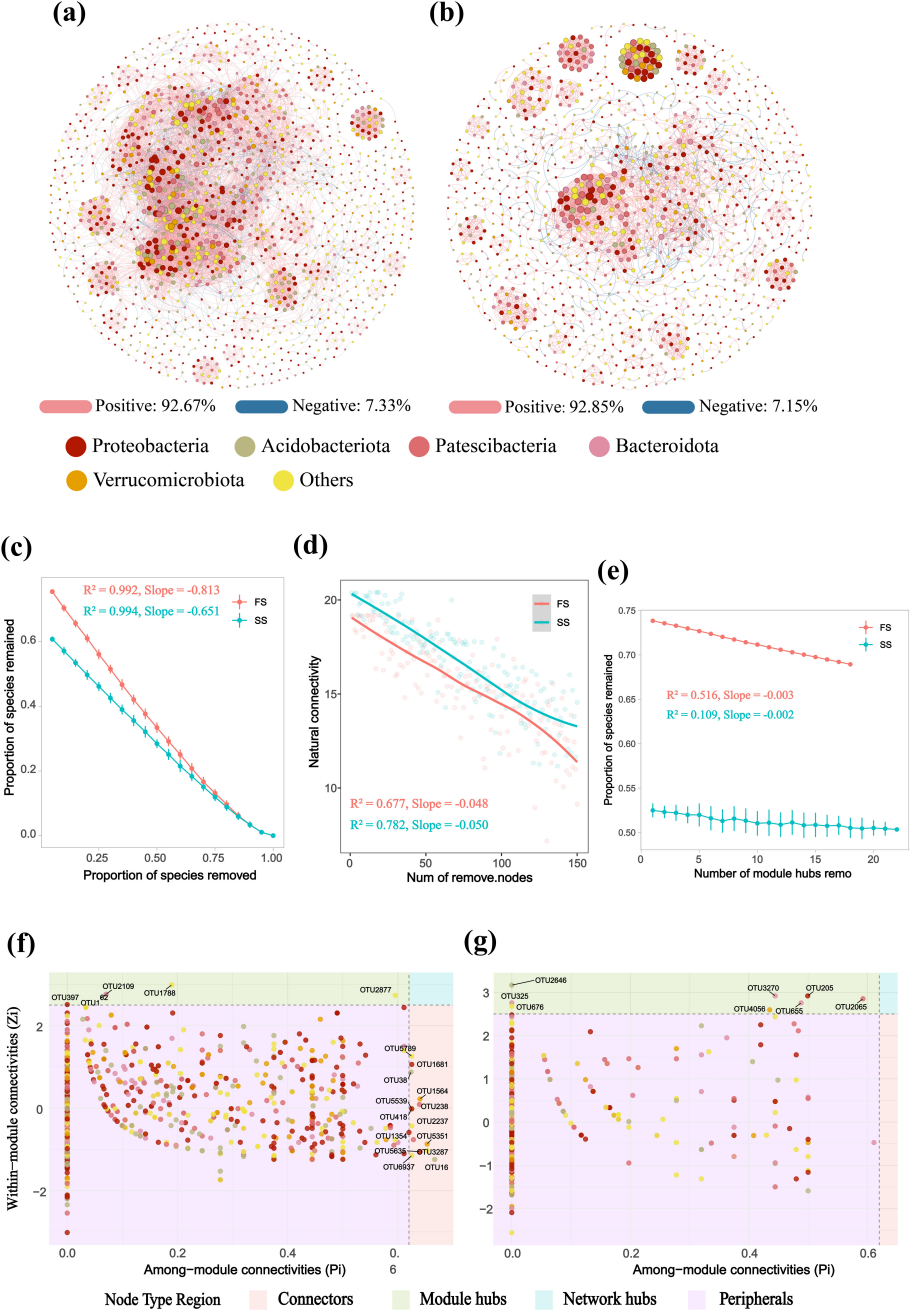
Rhizosphere microbial co-occurrence network and network stability analysis. **(a,b)** Microbial co-occurrence networks in the **(a)** FS and **(b)** SS. **(c)** Robustness analysis under random node removal. **(d)** Changes in the natural connectivity of microbial networks in the FS and SS during random species removal. **(e)** Impact of targeted attack on keystone nodes on network stability in the FS and SS. **(f,g)** Classification of keystone microbes in **(f)** FS and **(g)** SS. Keystone categories were defined based on within-module connectivity (Zi) and among-module connectivity (Pi): Peripherals (Zi ≤ 2.5 and Pi ≤ 0.62), Module hubs (Zi > 2.5 and Pi ≤ 0.62), Connectors (Zi ≤ 2.5 and Pi > 0.62), and Network hubs (Zi > 2.5 and Pi > 0.62). FS, Flowering Stage; SS, Senescing Stage.

We further assessed changes in the complexity of the rhizosphere microbial co-occurrence network between the FS and SS in *R. hybridum* via network analysis. Key topological parameters were calculated, including the number of nodes, number of links, average degree, network diameter, average clustering coefficient, and relative modularity. Compared to the FS, the SS network exhibited a significant reduction in both network scale (number of nodes: FS 1381 vs. SS 1141) and links (number of links: FS 11139 vs. SS 4351; average degree: FS 16.132 vs. SS 7.627). Although the network diameter was comparable (FS 17 vs. SS 16), indicating relatively stable global links, the SS network possessed a higher average clustering coefficient (FS 0.574 vs. SS 0.651), reflecting a structural shift toward local clustering. The FS network demonstrated higher relative modularity (RM: FS 3.087 vs. SS 2.608), revealing a more compartmentalized structure where the community was divided into more highly connected modules with sparse inter-module connections. In contrast, the low RM value of the SS network indicated a weakening of compartmentalization and a blurring of module boundaries. These results demonstrate that as plants entered the SS, both the overall complexity and the total number of links in the rhizosphere microbial interaction network decreased significantly. Notably, despite the reduction in total links, the average clustering coefficient increased concurrently. This suggests that the remaining interactions did not vanish randomly but became more concentrated and intensified within local microbial clusters. This reorganization, characterized by enhanced local clustering and weakened compartmentalization, signifies a structural transition of the network from the highly complex, modular architecture of the FS towards a simplified yet tightly interconnected configuration of local clusters in the SS (Table 1; Figures 4a,b).

**TABLE 1:** Topological properties of the rhizosphere microbial network.

Network	Nodes	Links	Average degree	Diameter	Average CC	RM
FS	1,381	11,139	16.132	17	0.574	3.087
SS	1,141	4,351	7.627	16	0.651	2.608

The total number of nodes; Links: the total number of links; Diameter: network diameter; Average CC, Average clustering coefficient; RM, Relative modularity; FS, Flowering Stage; SS, Senescing Stage.

We assessed changes in network stability across developmental stages by analyzing the robustness of the microbial co-occurrence network to random node removal, natural connectivity, and targeted attack on keystone nodes (Figures 4c–e). Analysis of random node removal robustness revealed that although the more complex FS network exhibited greater overall robustness, its stability decayed at a faster rate (slope: −0.813 vs. −0.651). In contrast, the structurally simplified SS network, while more fragile, demonstrated a more linear functional decline (Figure 4c). Natural connectivity analysis indicated a significantly higher degradation rate of topological stability in the SS network (slope: −0.050 vs. −0.048), suggesting that its sparsely connected structure disintegrated more rapidly upon species loss (Figure 4d). In the targeted attack on keystone nodes, the stability of the SS network showed a weak correlation with keystone nodes (*R*^2^ = 0.109) and declined at a very slow rate (slope = −0.002). This stood in sharp contrast to the FS network (*R*^2^ = 0.516, slope = −0.003). These results collectively indicate that the SS network possesses a decentralized topology, thereby conferring greater resilience against targeted attacks on keystone nodes (Figure 4e). These findings jointly demonstrate two key shifts driven by the progression from flowering to senescence. First, it drives structural simplification of the microbial network. Second, it shifts the network’s stability strategy from an “efficient yet potentially fragile” hub-dependent mode to a “broadly vulnerable yet gradually declining” decentralized mode.

The rhizosphere microbial network of *R. hybridum* exhibited a highly modular, hub-based structure during the FS, which degraded into a decentralized, simplified network in the SS. Keystone nodes within the microbial co-occurrence network were classified based on within-module connectivity (Zi) and among-module connectivity (Pi) (Guimerà and Amaral, 2005; Figures 4f,g). The results demonstrated the identification of 19 keystone microbes in the FS (comprising 5 module hubs and 14 connectors) (Figure 4f), whereas only 8 keystone microbes (all classified as module hubs) were identified in the SS (Figure 4g). Network hubs were not detected in either stage.

### 3.5 Interactions between root metabolites and the rhizosphere microbial community, and deterministic versus stochastic processes in microbiome assembly during flowering and senescence

**FIGURE 5:**
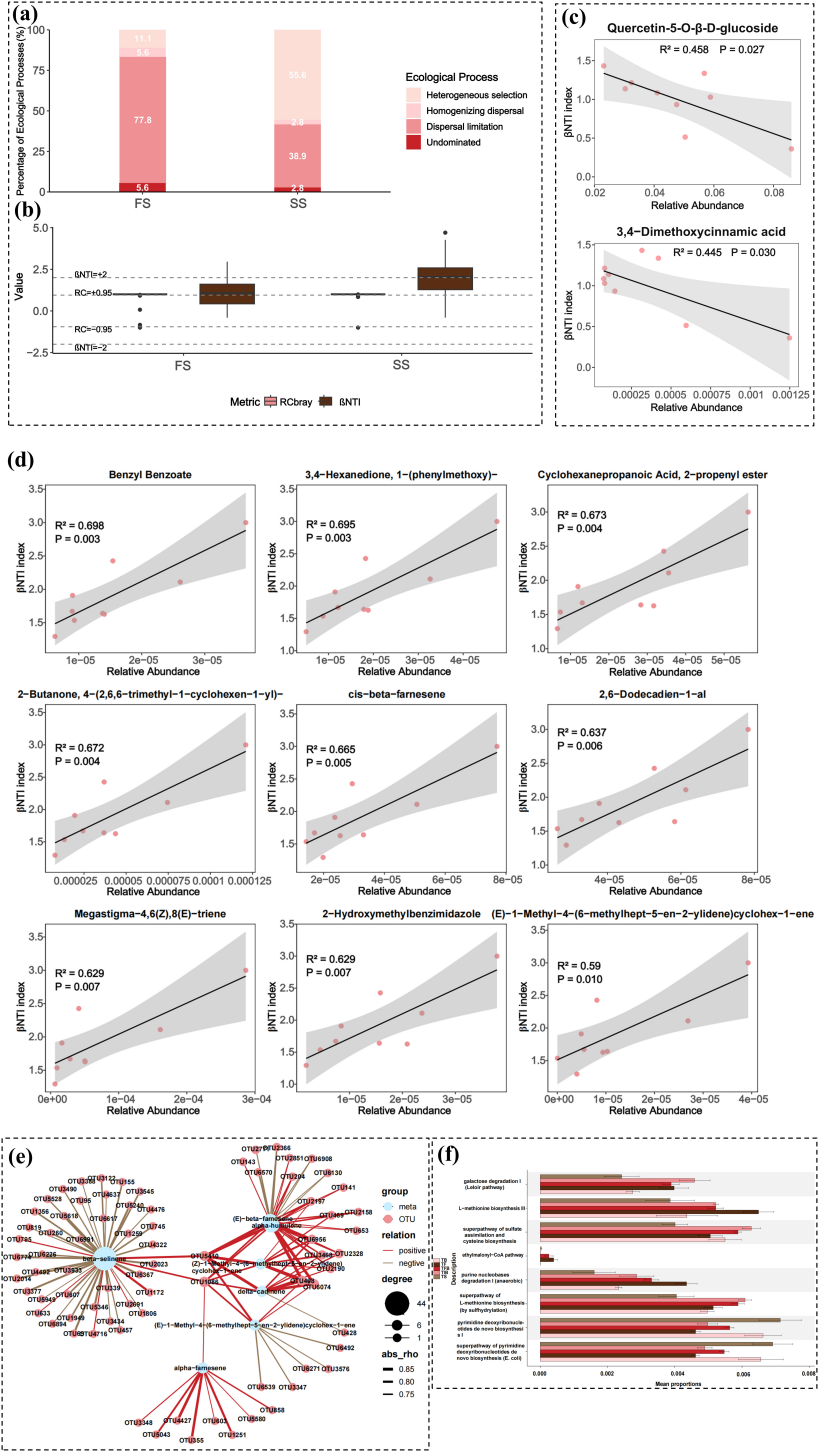
Analysis of deterministic and stochastic processes in microbial community assembly. **(a)** Stacked bar chart showing the relative contributions of ecological processes in the FS and SS. Community assembly mechanisms were categorized into five ecological processes based on βNTI (β-nearest taxon index) and RCbray (Raup-Crick index based on Bray-Curtis dissimilarity): heterogeneous selection (βNTI > +2), homogeneous selection (βNTI < - 2), dispersal limitation (|βNTI| < 2 and RCbray > +0.95), homogenizing dispersal (|βNTI| < 2 and RCbray < - 0.95), and undominated (|βNTI| < 2 and RCbray < 0.95). **(b)** Box plot comparing the relative contributions of deterministic (|βNTI| ≥ 2) and stochastic (|βNTI| < 2) processes in the FS and SS, based on |βNTI| values. **(c,d)** Linear regression analysis between root metabolite relative abundance and βNTI values in **(c)** FS and **(d)** SS. The adjusted R^2^ and *p*-value are annotated in the plots. **(e)** Correlation network between seven key metabolites and the rhizosphere microbial community. **(f)** Bar chart of significantly enriched metabolic pathways predicted by PICRUSt2 across stages TB to TF. TB, Bud Stage; TIB, Initial Blooming Stage; TFB, Full Bloom Stage; TS, Senescence Stage; TF, Fading Stage; FS, Flowering Stage; SS, Senescing Stage.

Plant root metabolites serve as both nutrients and signaling molecules, involved in the recruitment and structural remodeling of the rhizosphere microbial community. Root metabolites expressed at different developmental stages may regulate specific microbial taxa, thereby influencing plant growth and health (Feng et al., 2023; McLaughlin et al., 2023). Mantel tests indicated a significant correlation between root DAMs and rhizosphere microbial community structure during the flowering to senescence process (*p* = 0.002), demonstrating that root metabolites significantly influenced rhizosphere microbiome composition. To investigate the influence of root metabolites on microbial community assembly, we employed the βNTI and RCbray metrics to quantify the relative contributions of deterministic processes (including homogeneous selection and heterogeneous selection) and stochastic processes (including dispersal limitation, homogenizing dispersal, and drift). Null model analysis revealed significant differences in the dominant ecological processes governing rhizosphere microbiome assembly between the FS and SS. In the FS (TB, TIB, TFB), dispersal limitation was the dominant process, contributing 77.8%, followed by heterogeneous selection (11.1%). Upon transitioning to the SS (TFB, TS, TF), the proportion of heterogeneous selection increased to 55.6%, while dispersal limitation decreased to 38.9%, indicating that community assembly in this period was co-dominated by both deterministic and stochastic processes. The contributions of homogenizing dispersal and drift remained largely consistent across both stages (Figures 5a, b). Subsequently, correlation analysis between DAMs and βNTI was performed. In the FS, only 2 metabolites (quercetin-5-O-ß-D-glucoside and 3,4-dimethoxycinnamic acid) showed significant correlations with the microbial assembly process (Figure 5c). In contrast, in the SS, 109 metabolites exhibited significant associations, with the top 9 including benzyl benzoate, 3,4−Hexanedione, 1−(phenylmethoxy)-, and cyclohexanepropanoic acid, 2−propenyl ester (Figure 5d; Supplementary Table S7). This dramatic increase in the number of significantly correlated metabolites provides direct evidence that a large array of root-specific metabolites in the SS act as selective factors, regulating microbial survival and interactions. This result offers direct metabolomic evidence and suggests a potential chemical driving mechanism for the shift in community assembly processes—from predominantly stochastic processes (e.g., dispersal limitation) during the FS to predominantly deterministic processes (e.g., heterogeneous selection) during the senescent SS—as previously indicated by βNTI analysis. Collectively, these findings confirm that root metabolites play a central role in shaping the rhizosphere microbiome through a stage-dependent chemical regulation that transitions from weak to strong selective pressure.

To further elucidate the interactions between root metabolites and the rhizosphere microbiome during the transition from the FS to the SS in *R. hybridum*, we performed a correlation analysis between the 404 identified DAMs and 5,719 OTUs. The results revealed 25,056 significant correlation pairs (Supplementary Table S8), where the 404 DAMs were significantly correlated with 2,340 distinct OTUs, representing 44.84% of the total OTUs analyzed. KEGG enrichment analysis indicated that the DAMs were significantly enriched in the sesquiterpenoid and triterpenoid biosynthesis pathway (*p* < 0.05). Existing studies have shown that compounds from this pathway, such as SLs, jasmonates, and brassinosteroids, function as key endogenous signaling molecules. They integrate environmental signals with the plant’s internal status to coordinately regulate the flowering transition and senescence processes (Bai et al., 2024; Lu et al., 2025; Yin et al., 2025). SLs are also involved in regulating branching, leaf senescence, and root architecture, playing significant roles in plant development and nutrient acquisition (Zhou et al., 2025). Specifically, we identified 123 significant correlation pairs (70 positive and 53 negative) between 77 microbial taxa and 7 DAMs involved in sesquiterpenoid and triterpenoid biosynthetic pathways (Figure 5e).

PICRUSt2 was employed to predict the functional potential of the microbial community. We predicted the functional potential of 77 microorganisms significantly associated with seven DAMs in the biosynthesis pathways of sesquiterpenes and triterpenes (Supplementary Table S9). These microbes primarily belonged to families such as Chitinophagaceae, Acetobacteraceae, Rhodanobacteraceae, Comamonadaceae, and Bdellovibrionaceae, among others. Through PICRUSt2 functional prediction of these 77 microorganisms, we identified a series of statistically significantly enriched predicted metabolic pathways. These included galactose degradation I (Leloir pathway), L-methionine biosynthesis III, superpathway of sulfate assimilation and cysteine biosynthesis, ethylmalonyl-CoA pathway, purine nucleobases degradation I (anaerobic), superpathway of L-methionine biosynthesis (by sulfhydrylation), pyrimidine deoxyribonucleotides *de novo* biosynthesis I, and superpathway of pyrimidine deoxyribonucleotides *de novo* biosynthesis (*E. coli*) (Figure 5f). In summary, the functional prediction analysis highlighted the diversity of the rhizosphere microbial community involved in carbon metabolism and its potential roles in several crucial metabolic pathways, particularly in galactose degradation I (Leloir pathway), L-methionine biosynthesis III, and the superpathway of sulfate assimilation and cysteine biosynthesis.

## 4 Discussion

### 4.1 Dynamic changes in root metabolites from flowering to senescence and the key role of terpenoids

This study analyzed the dynamic changes in root metabolites during the flowering process of individual *R. hybridum* plants, revealing significant time-dependent alterations in metabolite abundance. This finding aligns with reported conclusions for *A*. *thaliana* developmental stages and the entire life cycle of rice, indicating that specific metabolic regulation plays an important role in plant development (Fan et al., 2025; Qian et al., 2023). Notably, root morphology and order (e.g., fine vs. coarse roots) can strongly influence exudation patterns and microbial recruitment (Herms et al., 2022; Pérez-Jaramillo et al., 2017). In this study, root samples were collected as bulk mixtures without distinguishing between different root orders (e.g., absorptive vs. transport roots). While this approach facilitated capturing the overall impact of plant developmental stage on the rhizosphere system, future investigations employing order-specific sampling would enable a more precise dissection of metabolite secretion and microbial colonization dynamics within specific rhizosphere micro-domains (e.g., fine root tips).

Our study found that the significantly changing DAMs in rhododendron roots were primarily enriched in the sesquiterpenoid and triterpenoid biosynthesis pathway. Existing research has shown that the rhizosphere microbiome structure of *A*. *thaliana* mutants defective in triterpene and diterpene biosynthesis differs significantly from the wild type, suggesting these compounds play a crucial role in rhizosphere microbial community assembly (Huang et al., 2019; Pang et al., 2021). Gao et al. (2025) reported a positive correlation between terpenoids and microbial diversity/community stability in *Codonopsis pilosula*. Furthermore, plants recruit beneficial microbes by secreting metabolites like sesquiterpenes, thereby enhancing resistance against pathogen invasion (Kulkarni et al., 2024). Within the sesquiterpenoid and triterpenoid biosynthesis pathway, seven volatile terpenoids—(E)-beta-farnesene, beta-selinene, alpha-humulene, delta-cadinene, alpha-farnesene, (Z)-1-Methyl-4-(6-methylhept-5-en-2-ylidene)cyclohex-1-ene, and (E)-1-Methyl-4-(6-methylhept-5-en-2-ylidene)cyclohex-1-ene—were significantly upregulated during the SS. Current research identifies (E)-beta-farnesene as possessing broad-spectrum antimicrobial activity, capable of inhibiting soil-borne pathogens and promoting the establishment of beneficial rhizosphere microbial communities (Henrique Fontoura et al., 2024). Beta-selinene, an inducible and constitutive anti-insect and antimicrobial metabolite, not only exhibits antifeedant or toxic effects on various insects but may also participate in shaping the rhizosphere microbial community structure (Danna et al., 2025; Miura et al., 2025). Similarly, Kong et al. (2021) reported that alpha-humulene can enhance plant tolerance to pathogens such as Acetobacter and Pseudomonas aeruginosa. The volatile sesquiterpenes alpha-farnesene and (E)-1-Methyl-4-(6-methylhept-5-en-2-ylidene)cyclohex-1-ene (also known as alpha-thujene) are also recognized for their key roles in plant growth and defense (Liao et al., 2022; Xu et al., 2025). Based on our results and existing literatures, we propose that metabolites in the root sesquiterpenoid and triterpenoid biosynthesis pathway undergo significant changes across different flowering stages in rhododendron. Specifically, during the SS, these volatile terpenoids likely function as a chemical barrier inhibiting pathogen invasion and influence microbial community structure through their selective antimicrobial activities. However, future studies should concurrently monitor the dynamics of rhizosphere soil physicochemical parameters (e.g., pH, available phosphorus, and ammonium nitrogen) and employ statistical modeling (e.g., variance partitioning analysis) to quantify the relative explanatory contributions of plant-derived specific metabolites versus traditional environmental factors to the microbial community. This would enable a more precise dissection of their driving mechanisms.

Our further investigation revealed that the seven volatile terpenoids—(E)-beta-farnesene, beta-selinene, alpha-humulene, delta-cadinene, alpha-farnesene, (Z)-1-Methyl-4-(6-methylhept-5-en-2-ylidene)cyclohex-1-ene, and (E)-1-Methyl-4-(6-methylhept-5-en-2-ylidene)cyclohex-1-ene—were significantly correlated with 77 rhizosphere microbial taxa during the transition from FS to SS. Notably, they showed significant positive correlations with four potential plant growth-promoting bacteria: *Bdellovibrio*, *Devosia*, *Dyella*, and *Rhodopila* (Supplementary Table S10). *Bdellovibrio* is a predatory bacterium possessing functions in both biological and abiotic control (Cavallo et al., 2021). Chhetri et al. (2022) demonstrated that *Devosi*a from the rice rhizosphere can synthesize the key plant auxin IAA in the presence of the precursor L-tryptophan. IAA effectively stimulates seed germination, root development, and proliferation, thereby enhancing the host’s ability to absorb water and nutrients. *Dyella* strains isolated from root nodules of Lespedeza sp. can produce IAA under *in vitro* conditions, while some strains also exhibit ACC deaminase activity, which can help plants reduce stress ethylene levels and enhance stress resistance (Palaniappan et al., 2010). *Rhodopila* is a phototrophic bacterium capable of photosynthesis under light, fixing CO_2_ and producing organic matter, potentially providing carbon sources to the rhizosphere (Tani et al., 2022). Functional prediction of the 77 rhizosphere microbes influenced by these terpenoid metabolites was performed using PICRUSt2, identifying a series of enriched metabolic pathways. These included galactose degradation I (Leloir pathway), L-methionine biosynthesis III, and the superpathway of sulfate assimilation and cysteine biosynthesis, among others. It should be emphasized that the microbial functions inferred by PICRUSt2 (e.g., methionine biosynthesis) represent phylogeny-based predictions. Although these predictions are highly consistent with the observed metabolite and community dynamics and have received partial support from the literature, they require empirical validation to confirm the functionality of the predicted pathways. The functional predictions revealed that the potential metabolic functions of this community strongly point toward the efficient utilization of root-secreted carbon sources. For instance, galactose degradation I (Leloir pathway) could provide the energy foundation for rapid microbial proliferation during the FS, aligning with the observed peak in microbial diversity at TFB (Dudko et al., 2023; Kareem et al., 2024). More interestingly, these microbes exhibited active sulfur assimilation and sulfur-containing amino acid synthesis capabilities. The enrichment of the methionine biosynthesis pathway is particularly noteworthy, as methionine is a key precursor for the plant hormone ethylene. This enrichment suggests a potential mechanism: the rhizosphere microbial community might indirectly influence ethylene biosynthesis in the plant by modulating the methionine supply in the rhizosphere, thereby potentially playing a regulatory role in the senescence process of rhododendron floral organs (Aragón-Raygoza and Strable, 2025; Chen et al., 2018). Furthermore, the robust nucleic acid metabolism—evidenced by pathways like de novo pyrimidine nucleotide biosynthesis and purine degradation/recycling—reflects high microbial proliferative activity during the FS (Katahira and Ashihara, 2002; Ouyang et al., 2025). These predicted functional potentials are highly consistent with our observations: both microbial α-diversity and network complexity peaked during the TFB. Active metabolism is the foundation for all ecological functions performed by the community. This indicates that throughout the flowering stages of rhododendron, sesquiterpenoids and triterpenoids are highly likely to be involved in enriching and regulating the composition of the rhizosphere microbial community. Furthermore, by influencing the abundance of rhizosphere plant growth-promoting bacteria, these compounds ultimately exert feedback effects on rhododendron growth and development, potentially fine-tuning the floral transition and senescence process through this intricate chemical dialogue.

### 4.2 Rhizosphere microbial community structure undergoes significant reorganization across distinct flowering stages

The rhizosphere microbial community experienced significant reorganization throughout the flowering process of *R*. *hybridum*, particularly between the FS and SS. PCoA indicated that 66.51% of the microbial community variation was attributable to floral stage succession, a finding further validated by a significant PERMANOVA result. Our results demonstrated that rhizosphere microbial richness and diversity peaked at the TFB but decreased significantly during the SS, suggesting that the FS provides a more favorable ecological environment for microbes. Beta diversity analysis further revealed a clear separation between TF and TFB samples (Figure 3a), indicating that each floral stage is associated with the specific enrichment or depletion of particular microbial taxa, consistent with reports by Xiong et al. (2021) and Zhalnina et al. (2018). At the phylum level, Acidobacteriota, Patescibacteria, and Bacteroidota were significantly enriched in the rhododendron rhizosphere (Figure 3h). The relative abundance of Acidobacteriota across the stages (TB to TF) exhibited a “U”-shaped pattern, whereas Patescibacteria and Bacteroidota showed an inverted “U”-shaped pattern (Supplementary Table S2). (Lu et al., 2018) found that these three phyla could be stably enriched across generations in the *A*. *thaliana* rhizosphere and are involved in regulating flowering time, suggesting their potential conserved role in microbe-host interactions governing developmental timing across diverse plant species.

As *R*. *hybridum* transitions from the FS to the SS, its rhizosphere microbial co-occurrence network undergoes significant structural reorganization, characterized by a decreased average degree, increased modularity, loss of keystone taxa, and a trend towards decentralization (Table 1; Figures 4a–b). Although the global connectivity (network diameter) remained largely unchanged, the weakening of inter-module connections and the rise in local clustering indicate an enhanced vulnerability in microbial interaction patterns and a reduction in network resilience. Keystone microbial taxa play a crucial role in maintaining community structure and functional stability (Du et al., 2025; Zhu et al., 2024). Through Zi-Pi analysis, this study identified 27 keystone microbes critical during the flowering and senescence stages (Figures 4f,g), most belonging to phyla such as Acidobacteriota, Proteobacteria, and Bacteroidota (Supplementary Table S11). Among them, the genus *Dongia* can participate in denitrification, thereby driving nitrogen transformation, and its abundance correlates positively with soil organic carbon and community assembly processes (Adamo et al., 2024; Shi et al., 2023). While the function of the genus *Puia* remains unclear, related studies suggest its potential role in assisting plant nutrient acquisition (Lv et al., 2017). *Gaiella* is closely associated with nitrogen and phosphorus cycling and can enhance plant stress resistance (Liu et al., 2023; Yang et al., 2023). *Pajaroellobacter* is linked to environmental adaptability (Baker et al., 2024). *Aquicella* can improve plant disease resistance; the *Burkholderia*-*Caballeronia*-*Paraburkholderia* cluster possesses multiple functions including enhanced thermotolerance, disease suppression, and associative nitrogen fixation (Cao et al., 2025; Liu et al., 2025); and *Occallatibacter* is involved in phosphate solubilization and nitrogen fixation processes (Wei et al., 2024). Compared to previous studies in the field, the keystone microbial taxa identified here (e.g., Acidobacteriota and Bacteroidota) have also been reported to be associated with developmental stages in model plants like *A*. *thaliana* and rice (Lu et al., 2018). In summary, these keystone taxa likely significantly influence plant growth and soil nutrient dynamics by participating in nitrogen and phosphorus cycling, enhancing plant stress resistance, promoting nutrient acquisition, and improving soil health. Furthermore, the presence of numerous unclassified taxa suggests that the rhododendron rhizosphere may harbor a vast reservoir of unexplored functional microbes, potentially playing specialized interactive roles during specific developmental stages such as TFB or TS. These results highlight the dynamic responsiveness of the microbial co-occurrence network throughout the plant life cycle and suggest that microbiome manipulation strategies targeting these keystone taxa could potentially enhance plant adaptability and ecological stability during the SS.

### 4.3 Root metabolites modulate rhizosphere microbiome assembly across distinct floral stages in rhododendron

The selective role of root metabolites in shaping rhizosphere microbial community assembly is receiving increasing attention. Research indicates that plants regulate the composition and function of the rhizosphere microbiome through developmental stage-dependent synthesis and secretion of metabolites (Baker et al., 2024; Santangeli et al., 2024). Our study found that in *R. hybridum*, significant changes in the abundance and composition of root metabolites during the transition from the FS to the SS likely directly drive the restructuring of the rhizosphere microbial community (Zhang et al., 2025). At the TFB, root metabolite diversity reached its peak. Concurrently, the α-diversity of the rhizosphere microbial community (e.g., Chao1 and Simpson indices) also peaked, suggesting that higher resource availability may support the coexistence of a richer diversity of microbial species and facilitate niche differentiation (Dominchin et al., 2025). Further analysis revealed significant correlations between DAMs and microbial community structure. Secondary metabolites such as terpenoids, flavonoids, and phenolic acids likely function as carbon sources or signaling molecules, directly regulating microbial growth and interactions (Li et al., 2025). Through database comparisons, we identified 36 potential rhizosphere beneficial bacterial taxa (Supplementary Table S12), which exhibited distinct enrichment patterns between the FS and SS stages. Furthermore, during FS, the rhizosphere microbial co-occurrence network displayed greater complexity and compartmentalization (Figure 4a). This pattern aligns with relatively abundant and homogeneous resource conditions, implying that plants may maintain higher microbial diversity during the reproductive phase to enhance the stability and resilience of the rhizosphere ecosystem (Caruso et al., 2011).

When plants enter the SS, their physiological focus shifts from reproduction to nutrient recycling, tissue degradation, and stress pre-adaptation. Consequently, the rhizosphere nutritional environment likely transitions from abundant to scarce or heterogeneous. This shift drives a change in the microbial community assembly mechanism, from a predominance of stochastic processes to one governed by deterministic processes, manifested specifically by a significant enhancement in the role of heterogeneous selection (Figure 5a). As noted by Feng et al. (2018), drastic changes in resource conditions often lead to deterministic processes dominating community differentiation. We posit that this strategic shift—from a “broad-spectrum recruitment” to a “targeted recruitment” approach—reflects an adaptive mechanism where plants actively screen for functional microorganisms via stage-specific root metabolites (e.g., certain volatile triterpenes). The biological essence of this “selective recruitment” likely involves multiple synergistic mechanisms: specific metabolites can act as selective antimicrobial agents (e.g., the SS upregulated (E)-beta-farnesene, whose broad-spectrum antibacterial activity may inhibit some microbes, reshaping the competitive landscape); as signaling molecules (e.g., terpenoids and flavonoids, potentially regulating chemotaxis, colonization, or gene expression in beneficial microbes); and as differential metabolic substrates (forming a unique nutritional matrix that supports the proliferation of microbes possessing corresponding metabolic pathways). The predicted microbial functions in SS, showing significant enrichment in sulfur metabolism and methionine biosynthesis pathways, provide an integrative perspective for this view: this enrichment may result from “nutrition-driven” selection via the secretion of specific substrates by the plant, and/or be associated with a “signaling-coordination” process where recruited microbes contribute to synthesizing ethylene precursors to regulate senescence.

In summary, the assembly process of the rhizosphere microbial community in *R. hybridum* undergoes a systematic transformation aligned with floral developmental stages. This transformation is not merely a passive response to changing rhizosphere resource conditions but likely represents an active microbial management strategy implemented by the plant to adapt to its intrinsic developmental program.

### 4.4 A dynamic interaction model between root metabolites and the rhizosphere microbiome during the flowering process of rhododendron

**FIGURE 6:**
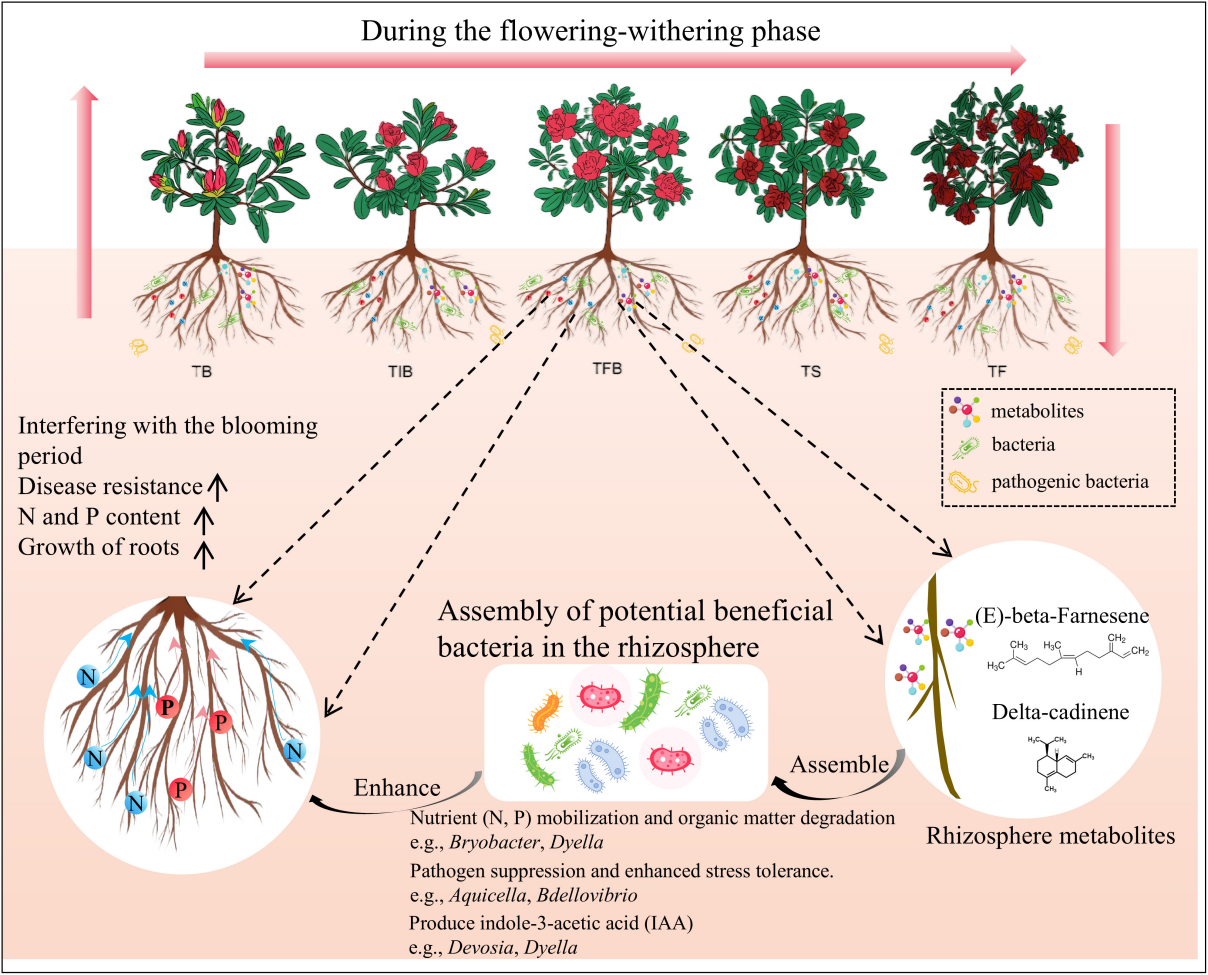
Dynamic interaction mechanism between metabolites and the rhizosphere microbiome during the flowering process of *Rhododendron*. TB, Bud Stage; TIB, Initial Blooming Stage; TFB, Full Bloom Stage; TS, Senescence Stage; TF, Fading Stage; FS, Flowering Stage; SS, Senescence Stage.

This study provides a preliminary exploration of the dynamic temporal changes in root metabolites and the rhizosphere microbiome, as well as their interaction mechanisms, during the FS and SS. Integrating our analytical results with existing research, we propose a dynamic interaction model for the flowering process in *Rhododendron* (Figure 6), positing that: (1) Both root metabolites and the rhizosphere microbiome undergo significant temporal dynamics across different flowering stages in *R*. *hybridum*, as evidenced by the STEM analysis of metabolite contents from TB to TF and the observed shifts in microbial community assembly between FS and SS. (2) Throughout the flowering process, the DAMs are predominantly enriched in the sesquiterpenoid and triterpenoid biosynthesis pathway. This specific enrichment is likely a result of selective pressure influenced by the floral stage. More importantly, these terpenoid metabolites can influence rhizosphere microbial community assembly and attract a substantial number of potential beneficial rhizobacteria, potentially exerting positive effects on plant growth, nutrient availability during flowering, and soil health. (3) The microbial taxa impacted during the flowering process, as indicated by literature review and functional prediction, possess capabilities such as nitrogen fixation, degradation of complex organic matter, and phosphorus solubilization. These functions are crucial for ensuring nutrient availability for the host plant during the FS and enhancing its resistance to biotic and abiotic stresses during the SS.

Collectively, through integrative multi-omics analyses, this study systematically reveals the coordinated temporal succession of both the rhizosphere microbial community and the metabolite profile in *R. hybridum* during the flowering-to-senescence transition. These correlative data lead us to propose a core mechanism-focused hypothesis: the enriched methionine biosynthesis pathway within the rhizosphere microbiome may serve as a precursor pool for ethylene, thereby indirectly regulating the plant senescence process. Notably, methionine is also a universal marker for fundamental microbial metabolism and growth. Therefore, the observed pathway enrichment could represent either a functional adaptation where microbes actively participate in plant hormone signaling or a metabolic byproduct stemming from the proliferation of specific taxa. To distinguish between these possibilities and establish causality, future validation should proceed along two parallel dimensions: first, employing synthetic communities (SynComs) and targeted cultivation in controlled systems to test the “microbe-methionine-plant ethylene” axis; second, combining metagenomics with isolation techniques to further resolve the specific functions of key taxa such as Acetobacteraceae and Chitinophagaceae. Additionally, the microbial interaction networks constructed here, based on *n* = 3 per stage, require confirmation of their robustness through resampling analyses with expanded sample sizes. Together, these systematic validation efforts will advance our understanding from correlative observations toward a mechanistic and causal comprehension of the rhizosphere microecological regulation governing floral transition in *Rhododendron*.

## 5 Conclusion

This study reveals that root metabolites, particularly sesquiterpenoids and triterpenoids, significantly influence the structure and assembly of the rhizosphere microbial community during the flowering-to-senescence process in *R*. *hybridum*. The results demonstrate that as the plant transitions from the FS to the SS, distinct changes in root metabolite abundance occur, which are closely associated with the dynamics, co-occurrence patterns, and assembly processes of the rhizosphere microbiome. Notably, we observed significant fluctuations in the levels of metabolites such as (E)-beta-farnesene, beta-selinene, alpha-humulene, delta-cadinene, and alpha-farnesene, which effectively enriched microbial taxa with potential plant-beneficial functions. Furthermore, we found that root metabolites significantly modulate the assembly of the rhizosphere microbial community. Based on functional prediction analysis, this study found that metabolite-influenced microbial communities exhibit potential functions such as active sulfur metabolism and methionine biosynthesis. This leads to a hypothesis worthy of further validation: these microbes may form functional coupling with plant senescence programs (e.g., ethylene synthesis) by participating in rhizosphere sulfur cycling and methionine metabolism. This provides key clues and a theoretical basis for subsequent exploration of whether and how the microbiome feedback-regulates plant development, and also offers potential strategies for delaying flower senescence and enhancing ornamental value through targeted regulation of the rhizosphere microbiome. It should be noted that the above functional predictions are based on the PICRUSt2 algorithm, and their accuracy is limited by the completeness of the database; therefore, the related interpretations remain a hypothetical framework. Future work should employ methods such as metagenomic sequencing, targeted metabolomics validation, and axenic system experiments to further confirm microbial functions and elucidate their causal interactions with plants.

In summary, through temporal correlation analysis of metabolomic and microbiome data, this study revealed significant correlations between root metabolites (especially triterpenoids) and the assembly dynamics of the rhizosphere microbial community during floral transition in *R. hybridum*. We further propose that triterpenoid-mediated microbiome restructuring may not only be a developmental stage-specific feature in *R. hybridum*, but also a conserved adaptive strategy widely adopted by plants of the genus *Rhododendron* during senescence, aimed at optimizing nutrient recycling and stress defense through precise regulation of functional rhizosphere microbes. This hypothesis and related functional inferences await further experimental validation through axenic systems, synthetic community inoculation, and cross-species comparative studies. This study provides an important correlational and hypothetical foundation for understanding the functional coupling mechanism between plant development and the rhizosphere microbiome.

## Data Availability

The datasets presented in this study can be found in NCBI online repositories. The names of the repositories and accession number(s) can be found at: https://www.ncbi.nlm.nih.gov/sra, PRJNA1359395.

## Footnotes

1
https://new.metaboanalyst.ca/

2
https://www.omicshare.com/tools/

3
https://www.omicstudio.cn/tool
