# Supplementary material for: Root triterpenoid metabolites drive the assembly and feedback regulation of the rhizosphere microbiome during flowering to senescence in Rhododendron hybridum ‘Yangmeihong’
Source: Front Microbiol. 2026 Feb 10;17:1753104. doi: 10.3389/fmicb.2026.1753104 (PMC12931525; doi:10.3389/fmicb.2026.1753104)
Supplement: Supplementary file 1 [file Data_Sheet_1.pdf]

Figure S1. Samples from the bud stage to the fading stage: (A) whole plant samples and (B) petal samples. TB, Bud Stage; TIB, Initial Blooming Stage; TFB, Full Bloom Stage; TS, Senescence Stage; TF, Fading Stage.

Figure S2. Mass Spectrometry Analysis of Secondary Metabolites. (A) Mass spectrometry detection spectrum of secondary metabolites in negative ion mode. (B) Mass spectrometry detection spectrum of secondary metabolites in positive ion mode. (C) Overlay of total ion chromatograms (TICs) for quality control (QC) samples of volatile metabolites, reflecting the stability of chromatographic separation and detection.

Figure S3. Root Metabolite Analysis. (A) KEGG pathway enrichment analysis of the 404 common differentially accumulated metabolites identified across six comparison groups during the flowering to senescence process (TB vs. TIB, TIB vs. TFB, TB vs. TFB, TFB vs. TS, TS vs. TF, TFB vs. TF). The top 10 most significantly enriched pathways, ranked by Q value (FDR-corrected p-value), are displayed. The x-axis represents $-\log_{10}$ (Q value), and the y-axis shows the pathway names. (B) Pie chart showing the distribution of the significantly differentially accumulated metabolites across 20 distinct classes. TB, Bud Stage; TIB, Initial Blooming Stage; TFB, Full Bloom Stage; TS, Senescence Stage; TF, Fading Stage.

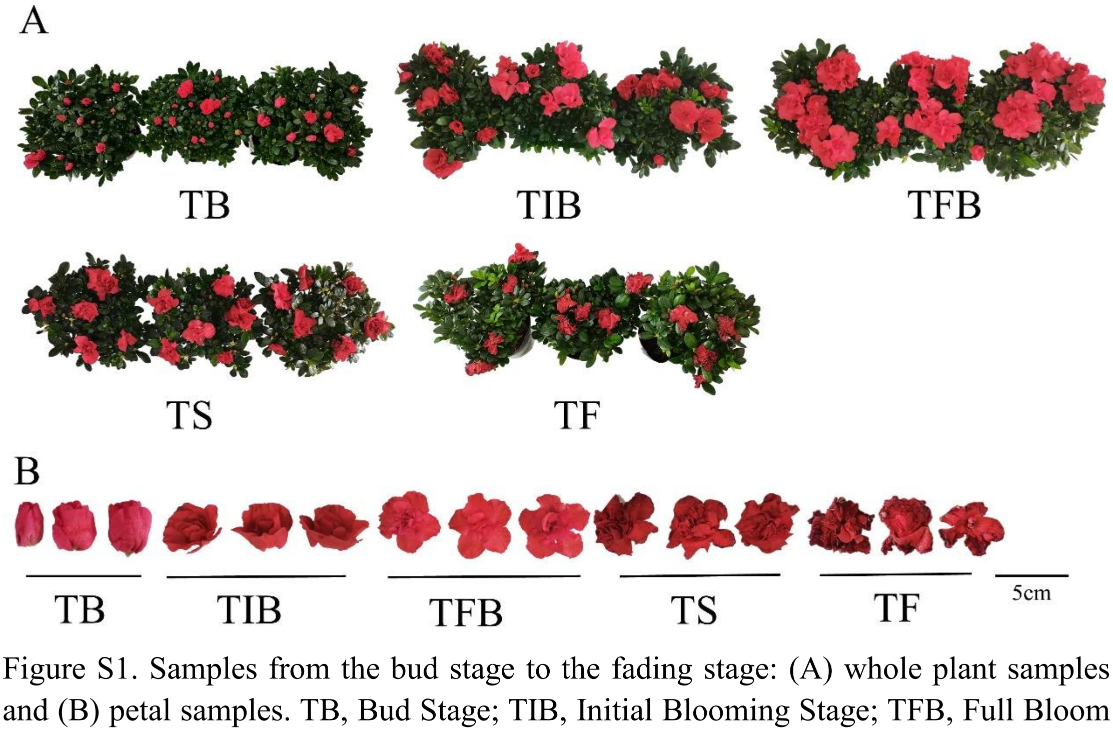

Figure S1. Samples from the bud stage to the fading stage: (A) whole plant samples and (B) petal samples. TB, Bud Stage; TIB, Initial Blooming Stage; TFB, Full Bloom Stage; TS, Senescence Stage; TF, Fading Stage.

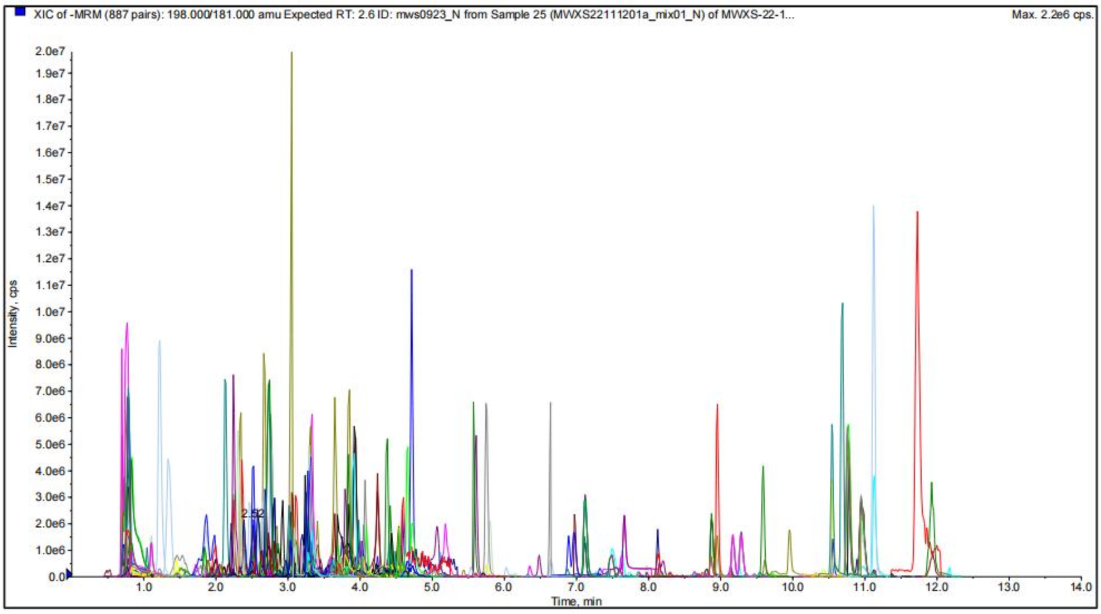
XIC of -MRM (887 pairs): 198.000/181.000 amu Expected RT: 2.6 ID: mws0923_N from Sample 25 (MWXS22111201a_mix01_N) of MWXS-22-1...
Max. 2.2e6 cps.
Intensity, cps
Time, min
2.52
0.0
1.0e6
2.0e6
3.0e6
4.0e6
5.0e6
6.0e6
7.0e6
8.0e6
9.0e6
1.0e7
1.1e7
1.2e7
1.3e7
1.4e7
1.5e7
1.6e7
1.7e7
1.8e7
1.9e7
2.0e7
1.0
2.0
3.0
4.0
5.0
6.0
7.0
8.0
9.0
10.0
11.0
12.0
13.0
14.0

（A）

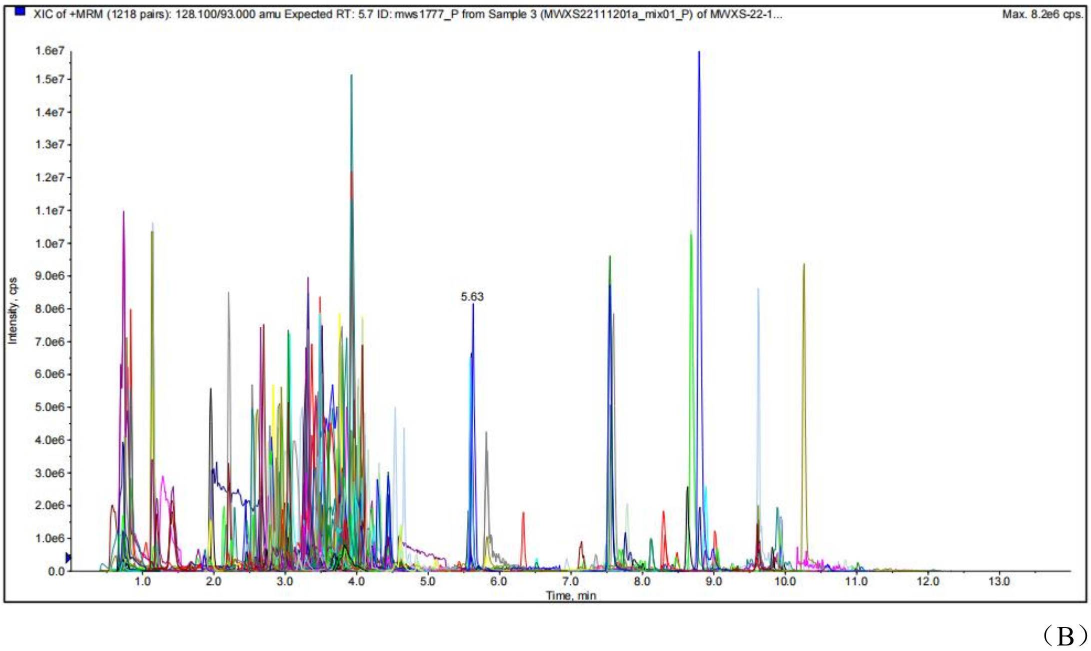
XIC of +MRM (1218 pairs): 128.100/93.000 amu Expected RT: 5.7 ID: mws1777_P from Sample 3 (MWXS22111201a_mix01_P) of MWXS-22-1...
Max. 8.2e6 cps.
Intensity, cps
Time, min
5.63
0.0
1.0e6
2.0e6
3.0e6
4.0e6
5.0e6
6.0e6
7.0e6
8.0e6
9.0e6
1.0e7
1.1e7
1.2e7
1.3e7
1.4e7
1.5e7
1.6e7
1.0
2.0
3.0
4.0
5.0
6.0
7.0
8.0
9.0
10.0
11.0
12.0
13.0

（B）

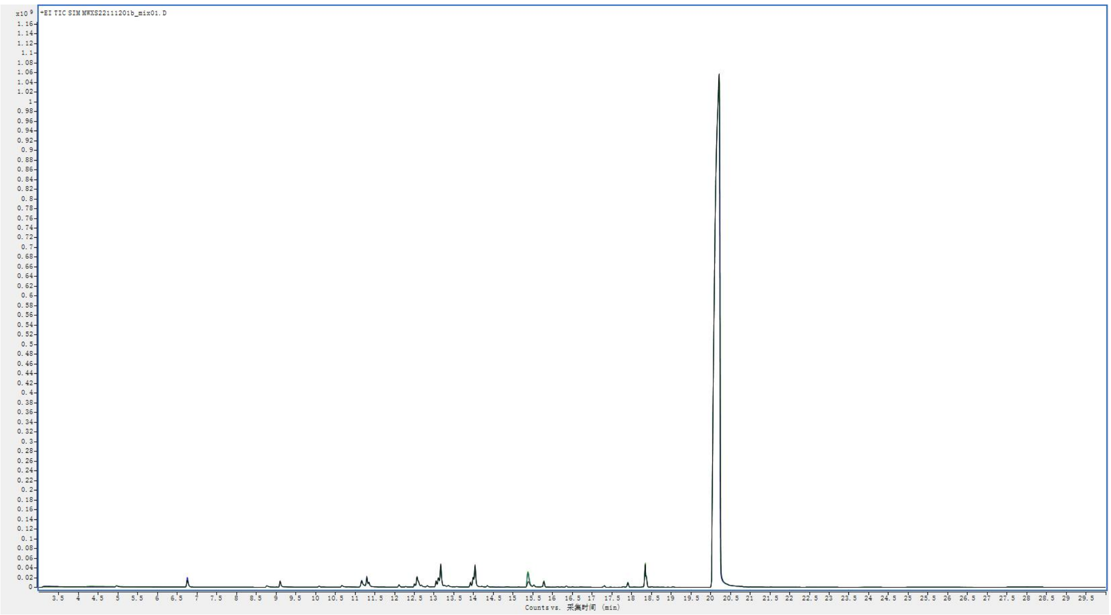

Figure S2. Mass Spectrometry Analysis of Secondary Metabolites. (A) Mass spectrometry detection spectrum of secondary metabolites in negative ion mode. (B) Mass spectrometry detection spectrum of secondary metabolites in positive ion mode. (C) Overlay of total ion chromatograms (TICs) for quality control (QC) samples of volatile metabolites, reflecting the stability of chromatographic separation and detection.

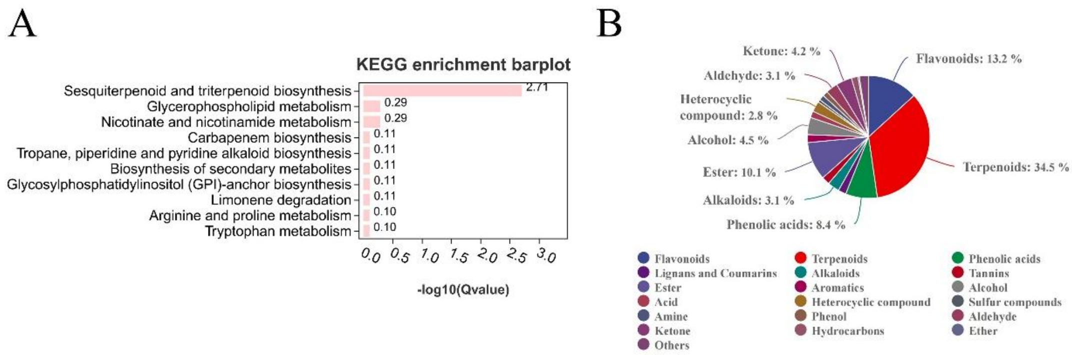

Figure S3. Root Metabolite Analysis. (A) KEGG pathway enrichment analysis of the 404 common differentially accumulated metabolites identified across six comparison groups during the flowering to senescence process (TB vs. TIB, TIB vs. TFB, TB vs. TFB, TFB vs. TS, TS vs. TF, TFB vs. TF). The top 10 most significantly enriched pathways, ranked by Q value (FDR-corrected p-value), are displayed. The x-axis represents -log$_{10}$ (Q value), and the y-axis shows the pathway names. (B) Pie chart showing the distribution of the significantly differentially accumulated metabolites across 20 distinct classes. TB, Bud Stage; TIB, Initial Blooming Stage; TFB, Full Bloom Stage; TS, Senescence Stage; TF, Fading Stage.
